# A cross-sectional study on peripheral arterial disease in a district of Sri Lanka: prevalence and associated factors

**DOI:** 10.1186/s12889-015-2174-7

**Published:** 2015-08-28

**Authors:** Janaka Weragoda, Rohini Seneviratne, Manuj C. Weerasinghe, Mandika Wijeyaratne, Anil Samaranayaka

**Affiliations:** Ministry of Health, Colombo, Sri Lanka; Department of Community Medicine, Faculty of Medicine, University of Colombo, Colombo, Sri Lanka; Department of Surgery, Faculty of Medicine, University of Colombo, Colombo, Sri Lanka

## Abstract

**Background:**

Peripheral arterial disease (PAD), a slowly progressive atherosclerotic disease affecting vital organs of the body, is increasingly recognized as a health burden worldwide. Epidemiological information on peripheral arterial disease is scarce in Sri Lanka. The present study intended to estimate the prevalence and associated factors of PAD among adults aged 40–74 years in Gampaha district, Sri Lanka.

**Methods:**

A cross-sectional study was carried out to estimate the prevalence of PAD among adults aged 40–74 years in four randomly selected divisional secretariat areas in Gampaha district in 2012–2013. The sample size of 2912 adults was obtained from 104 clusters using multistage probability proportionate to size sampling. The number of individuals to be included in the 5-year age groups between 40 and 74 years was determined based on the population proportion of the respective age groups in the district. Cluster size was 28, and equal numbers of males and females were selected for each age group per cluster. PAD was defined as having an ankle-brachial pressure index ≤ 0.89.

**Results:**

The age-and sex-standardized prevalence of PAD, adjusted for the sensitivity of the ankle-brachial pressure index was 3.6 % (95 % CI 2.9–4.3 %), and no significant difference was found between males (3.7 %) and females (3.6 %) (*p* = 0.08). Eighty-eight individuals were newly identified as having PAD, and a significant trend of prevalence with increasing age was observed (*p* < 0.001). Histories of diabetes mellitus, hypertension, dyslipidemia, coronary artery disease, cerebrovascular accident, smoking, and erectile dysfunction among males were significantly associated with PAD (*p* <0.001). Only one third of those with PAD experienced claudication symptoms.

**Conclusions:**

PAD was found to be a hidden disease in the Gampaha district population. Although there is minimal attention on PAD at present, the disease is likely to become a problematic public health concern in Sri Lanka, particularly with its aging population. Primary prevention measures to modify risk factors of PAD, including screening activities for early identification, should be a priority.

## Background

Peripheral Arterial Disease (PAD) is a slowly progressive atherosclerotic disease usually characterized by occlusion of lower limb arteries, ultimately causing acute or chronic limb ischemia [1, 2]. It is the third most important atherosclerotic disease after coronary artery disease and cerebrovascular disease [3] and is increasingly recognized as a health burden worldwide [1].

The prevalence of PAD in European and Asian populations is 3–10 % among those aged 40–70 years, and 10–20 % among those over 70 years of age [4–8]. The ankle-brachial pressure index (ABPI) is a simple, inexpensive, and noninvasive test that provides objective measurements. It can be easily performed in field epidemiological surveys, in clinical practice, and in vascular laboratories to diagnose PAD. The ABPI is defined as the ratio of the highest ankle systolic blood pressure divided by the highest brachial systolic blood pressure [9–12].

In Sri Lanka, data on PAD are scarce. Anecdotal evidence suggests that PAD has been detected in large numbers of patients in late stages of severe limb ischemia or ischemic leg ulceration. PAD has not yet, however, emerged as a public health problem in Sri Lanka, possibly because of a lack of data. The present study intended to estimate the prevalence and associated factors of PAD among adults aged 40–74 years in Gampaha District, Sri Lanka.

## Methods

**Fig. 1:**
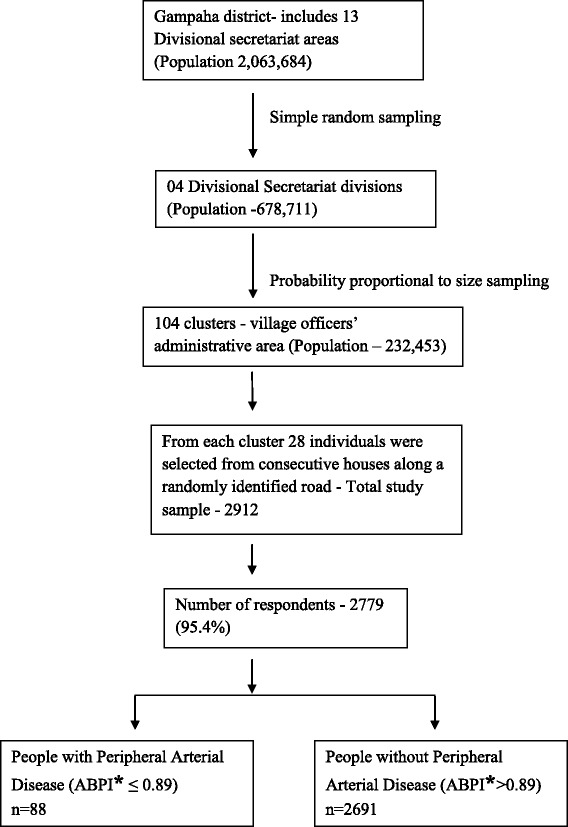
Schematic presentation of the study framework

We employed a cross-sectional study design to estimate the prevalence of PAD among adults aged 40–74 years in four randomly selected divisional secretariat areas in Gampaha District, Sri Lanka in 2012–2013. The district has been divided into 13 divisional secretariat areas for administrative convenience. The sample size of 2912 adults, aged 40–74 years, was obtained from 104 clusters using multistage probability proportionate to size sampling. A cluster was defined as an administrative area of a village officer and clusters size was 28 (Fig. 1). Administrative area of a village officer has a population that ranges from 1000–3000. The proportion of population between ages 40–74 years is around 27 %. Sample was obtained from each cluster in 5- year age category of 40–44, 45–49, 50–54, 55–59, 60–64, 65–69, 70–74 years and the number of individuals to be included in different age category was determined based on the population proportion of the respective age categories. Equal number of males and females were selected for each age category per cluster. The population proportion of 5-year age groups in the district was obtained from 2001 census data.

The sample size was determine using the formula for prevalence study described in Lwanga and Lemeshow [13]. The critical value of specified confidence interval of 95 % is 1.96. Anticipated population prevalence of PAD was taken as 3.5 % and the absolute precision required on either side of the proportion (1.0 %). Since cluster sampling method was adopted the effect of clustering was overcome by making a correction for design effect. The design effect was taken as 2.0. An additional 12 % was added to account for non response among participants. Thus, the minimum sample size required to detect the expected prevalence of PAD in the community with 95 % confidence interval and with 1 % precision was 2912.

A prevalidated, interviewer-administered questionnaire was used to obtain data. Socio-demographic characteristics such as age, sex, ethnicity, religion, level of education, and monthly household income were obtained. The age was verified by supportive documents. Medical history and duration of diabetes mellitus, dyslipidemia, hypertension, coronary arterial disease (CAD), cerebrovascular disease (CVD), and PAD was based on self-reporting and was verified either by available clinical records or medications.

The assessment of intermittent claudication was based on the Edinburgh claudication questionnaire [14]. Erectile dysfunction (ED) was defined as the recurrent or persistent inability to attain and/or maintain an erection for satisfactory sexual performance [15] and the information on ED was obtained using a selected set of questions.

Lifestyle characteristics of smoking and usage of alcohol were also obtained. Smoking exposure was categorized according to the classification of the Centers for Disease Control and Prevention in the United States [16]. Lifetime exposure to smoking was assessed by pack-year smoking values. Pack-years of smoking were calculated by multiplying the average number of cigarettes smoked per day by the number of years of smoking, then dividing by 20. Alcohol intake was categorized according to the definition of the National Institute on Alcohol Abuse and Alcoholism in United States [17]. Body weight was measured in kilogram to the nearest 0.1 kg using a “Seca 876” electronic digital standing-on weighing scale and the standing body height was measured in centimeter to the nearest 0.5 cm using a “Seca 213” stadiometer. Wight and height measurements were obtained according the guidelines given by Anthropometry Procedures Manual of Center for Disease Control and Prevention in the United States [18]. The categorization of body mass index (BMI) for obesity was based on the guidelines given by the International Obesity Task Force-WHO for Asians [19]. Each lower limb of the participants was examined for the status of pedal pulses; dorsalis pedis, and posterior tibial and categorized as present, weak or absent pulses. In addition, lower extremities were examined for signs of chronic circulatory insufficiency such as absence of hair growth, skin discoloration, dystrophic toe nails, fissured skin, ulceration, or gangrene. severity of ischemia of individuals with PAD was classified according to Fontaine’s stages [12].

The measurement of ABPI was performed according to the procedure described in the American College of Cardiology and the American Heart Association guidelines for the management of patients with peripheral arterial disease [12]. The Summit Vista ABI L450 arterial Doppler instrument made in United States was used to assess the ABPI. The ABPI was calculated up to two decimal places for each lower limb as the ratio of the highest systolic blood pressures at the ankle to the highest of the left and right brachial systolic pressures. The validity of ABPI to identify PAD was assessed using patients referred to the vascular laboratory at the National Hospital Sri Lanka (published separately). Those who were found with an ABPI of 0.89 or less in either lower limb were identified as having PAD. For ABPI ≤0.89, the sensitivity of detecting PAD was 87 %, and specificity was 99.1 %.

To standardize the performance of ABPI measurements, the principal investigator was a medical doctor trained under the guidance of a consultant vascular surgeon in National Hospital Sri Lanka. The Ethics Review Committee of the Faculty of Medicine, University of Colombo granted the approval for the study. Informed consent was obtained from all patients prior to participation.

Statistical Package for Social Science (SSPS) was used for analysis. The gross prevalence of PAD was standardized for age and sex using 2011 Sri Lankan census data and was also adjusted for ABPI score sensitivity. Bivariate analysis, followed by a multiple logistic regression, was conducted to assess the relationships of demographics, socioeconomic characteristics, and selected medical conditions with PAD.

## Results

The total number of participants was 2779, with a response rate of 95.4 %. The mean age of those with PAD was 64.3 years (SD = 7.59); median age was 65 years (IQR 60–70 years), with a range of 48–74 years.

All those found with PAD in this study (*n* = 88) were newly diagnosed with the disease. The crude prevalence was 3.16 %, and the age- and sex-standardized prevalence was 3.13 %. After adjusting for ABPI sensitivity, the estimated prevalence of PAD among the 40–74 years age group was 3.6 % (95 % CI2.9–4.3 %). The adjusted prevalence of PAD for males (3.7 %, 95 % CI2.7–4.7 %) and females (3.6 %, 95 % CI2.7–4.5 %) was not significantly different (*p* = 0.8). Table 1 shows the age-specific prevalence of PAD. No cases were found below the age of 45 years. A statistically significant trend (*p* < 0.001) of prevalence with increasing age was additionally discovered.

**Table 1:** Age- and sex-standardized prevalence of peripheral arterial disease by age group (adjusted for the sensitivity of the Ankle Brachial Pressure Index)

Age group (years)	40–44	45–49	50–54	55–59	60–64	65–69	70–74	Total
Prevalence	0 %	1.2 %	2.3 %	2.6 %	4.9 %	11.2 %	15.6 %	3.6 %

The demographic and socioeconomic characteristics of those found with PAD are described in Table 2. There were no significant differences in the prevalence of PAD among ethnic groups or between urban and rural sectors. A significantly higher prevalence of PAD was found among Christians (5.5 %), those with lower levels of education (4.6 %), and low-income groups (4.4 %) (*p* < 0.01).

**Table 2:** Participants’ sociodemographic characteristics by presence of PAD

Characteristics	PAD (*n* = 88)		No PAD (*n* = 2691)		Significance
	N	%	N	%	
Sex					
Male	45	3.3	1338	96.7	*p* = 0.8
Female	43	3.1	1353	96.9	
Sector					
Rural	65	2.9	2163	97.1	*p* = 0.1
Urban	23	4.1	528	95.9	
Ethnicity					*p* = 0.9
Sinhala	86	3.2	2615	96.8	
Others*	02	2.6	76	97.4	
Religion					*p* < 0.05
Buddhist	66	2.8	2255	97.2	
Christians	22	5.5	378	94.5	
Level of education					*p* < 0.01
GCE O/L not completed	39	4.6	853	95.4	
GCE O/L completed and above	49	2.7	1838	97.3	
Monthly family income Rs:					*p* < 0.01
<30,000	56	4.4	1227	95.6	
≥30,000 -	32	2.1	1464	97.9	

*Tamils and Muslims; GCE O/L: General certificate of education ordinary level

Compared to those without PAD, a significantly higher proportion of those with PAD had histories of diabetes mellitus (71.6 %), hypertension (78.5 %), dyslipidemia (73.9 %), CAD (15.9 %), CVD (11.4 %), and ED (62.2 %) (*p* < 0.001). Compared with those without PAD, a significantly higher proportion of those with PAD had diabetes mellitus (54.5 %), hypertension (56.7 %), or dyslipidemia (44.3 %) for 10 years or more (*p* < 0.01). A significantly higher proportion of males with PAD was found with erectile dysfunction (62.2 %) than those without PAD (*p* < 0.01). No significant difference was found in BMI and the usage of alcohol between those with and those without PAD (*p* > 0.05). No female smokers took part in the survey. Among males, there was a significantly higher proportion of smokers with PAD than without PAD (*p* < 0.001).A statistically significant trend in PAD occurrence was observed with higher exposure to pack-year smoking (*p* < 0.001) (Table 3).

**Table 3:** Selected characteristics among participants by presence of PAD

Characteristics	PAD		No PAD		Significance
	(*n* = 88)		(*n* = 2691)		
	N	%	N	%	
History of diabetes mellitus (yes)	63	71.6	464	17.2	*p* < 0.01
History of diabetes < 5 years	2	2.3	245	9.1	
History of diabetes 5–10 years	13	14.8	129	4.8	
History of diabetes 10 years	48	54.5	90	3.3	
History of hypertension (yes)	69	78.5	537	19.9	*p* < 0.01
History of hypertension < 5 years	5	5.7	352	13.1	
History of hypertension 5–10 years	15	17.0	120	4.4	
History of hypertension 10 years	49	56.7	65	2.4	
History of dyslipidemia (yes)	65	73.9	433	16.1	*p* < 0.01
History of dyslipidemia < 5 years	4	4.5	340	12.6	
History of dyslipidemia 5–10 years	22	25.0	65	2.4	
History of dyslipidemia 10 years	39	44.3	28	1.1	
History of Coronary artery disease (Yes)	14	15.9	85	3.2	*p* < 0.01
History of Cerebrovascular disease (Yes)	10	11.4	37	1.4	*p* < 0.01
Presence of Intermittent claudication (Yes)	33	37.5	42	1.5	*p* < 0.01
Erectile dysfunction among males* (Yes)	28	62.2	281	21.0	*p* < 0.01
Body mass index kg/m^2^					
<23	43	51.1	1075	38.1	df = 2
23–24.9	18	20.5	567	21.1	
≥25	27	28.4	1049	40.8	*p* > 0.05
Alcohol consumption					df = 2
Abstainers	45	51.1	1555	57.8	
Less frequent users	29	33.0	639	23.7	*p* > 0.05
Frequent users	14	15.9	497	18.5	
Smoking status	PAD (*n* = 45)		No PAD (*n* = 1338)		
Never smoker	06	13.3	645		df = 2 *p* < 0.001
Current smokers	20	44.4	367		
Former smokers	19	42.2	326		
Pack year smoking					df = 4
Zero (Never smokers)	6	13.3	645		
<5	4	8.9	328		
5-	4	8.9	174		*p* < 0.001
10 -	9	20.0	123		
≥20	22	48.9	68		

There were 130 affected lower limbs among the 88 individuals identified with PAD. Both pedal pulses were absent in 63.9 % of lower limbs with PAD. None of those affected lower limbs had a single normal pedal pulse. Skin manifestations of chronic circulatory insufficiency were found only in two third of the affected lower limbs (Table 4). Majority of those with PAD (62.5 %) belong to Fontaine’s stage I or asymptomatic and rest of the individuals (37.5 %) were belong to Fontaine’s stage II. None of those with PAD found with ischemic rest pain, ulceration or gangrene (Table 5).

**Table 4:** Status of pedal pulses and features of chronic circulatory insufficiency of lower limbs with and without PAD (*n* = 5558)

Status of pedal pulses	PAD		No PAD	
	(*n* = 130)		(*n* = 5428)	
(Dorsalispedis and Posterior tibial pulse)	No.	%	No	%
Both pedal pulse absent	83	63.9	--	--
Both pedal pulse diminished	25	19.2	93	1.7
One absent pulse with other diminished	22	16.9	114	2.1
One absent pulse and other normal	--	--	147	2.7
Both pulses normal	--	--	5074	93.5
Signs of chronic circulatory insufficiency^a^				
No foot signs	42	32.3	4918	90.6
Skin discoloration	58	44.6	258	4.8
Absent hair	44	33.8	89	1.6
Dystrophic nails	34	26.1	112	2.1
Fissured skin	16	12.3	51	0.9
Ulceration or gangrene	00	---	00	--

*n* number of lower limbs; ^a^One foot may have more than one sign

**Table 5:** Severity of the ischemia of those with PAD according to fontaine’s stages

Grade	Symptoms	Number	Percent
I	Asymptomatic	55	62.5
IIa	Mild claudication	15	17.0
IIb	Moderate – sever claudication	18	20.5
III	Ischemic rest pain	00	-
IV	Ulceration or gangrene	00	-

In logistic regression analysis, 5 years or more of diabetes mellitus, hypertension or dyslipidemia was significantly associated with PAD. The presence of myocardial infarction, CVA, and exposure to 10 or more pack-years of smoking were all also significantly associated with PAD (Table 6).

**Table 6:** Results of logistic regression analysis for factors associated with PAD

Factor	Significance
Level of education	.610
Monthly family Income	.342
Diabetes mellitus less than 5 years	.827
Diabetes mellitus 5–9 years	.002
Diabetes mellitus 10 years or more	.000
Hypertension less than 5 years	.246
Hypertension 5–9 years	.001
Hypertension 10 years or more	.000
Dyslipidemia less than 5 years	.080
Dyslipidemia 5–9 years	.000
Dyslipidemia 10 years or more	.000
Presence of myocardial infarction	.043
Presence of cerebrovascular accident	.021
Pack years smoking less than 10	.053
Pack year smoking 10 or more	.002

## Discussion

This study was the first community-based prevalence study on PAD in Sri Lanka and it was carried out in the Gampaha district. Epidemiological data related to PAD is scarce in Sri Lanka, and routine health information systems do not collect morbidity and mortality information directly related to PAD. Hence, the findings of this study will be useful for healthcare providers to estimate the burden of disease in the country and to plan screening programs for PAD.

A multistage probability proportionate to size cluster sampling method was used to obtain a representative sample from the district. There was high response rate of 95.4 %. The estimated prevalence of PAD among the age group of 40–74 years was 3.6 % (95 % CI 2.9–4.3 %). The age specific prevalence of PAD showed a consistent increase with advancing age, and there was no significant difference in prevalence between males and females. An urban population study in Chennai, India found a 3.2 % prevalence of PAD in an urban south Indian population [5]. The prevalence of PAD reported in a community-based study among South Asian migrants above the age of 45 years in the UK was 13.2 % (95 % CI 9.7–16.7) [20]. The prevalence of PAD reported in the present study is lower than that reported in studies from European countries and from the United States [21–23]. However, there is consistent evidence to support an age-related increase in trend [6–8]. Literature reveals no consistent evidence to support differences in the prevalence of PAD between the sexes. Some studies have found a significant difference in the prevalence between males and females [4, 21], while others have not [8, 20, 22]. In the present study, all those found with PAD were newly diagnosed with the disease. In one Barcelona study, 19 % of those found with PAD had been diagnosed previously [21]. This finding indicates the high need for a screening program in Sri Lanka for early detection and prevention of PAD complications.

The present study found a significantly higher proportion of individuals with diabetes mellitus, hypertension, and dyslipidemia among those with PAD than among those without PAD. This tendency has been consistently supported by other studies [4, 24, 25]. In many studies, the association of PAD has been assessed only in relation to the presence or absence-and not the duration—of these medical conditions. In our study, however, a 5-year or more duration of diabetes mellitus, dyslipidemia, or hypertension was found to be significantly associated with PAD. Although it is difficult to determine the exact time of onset of these diseases, the known duration of disease provides important information about development of PAD. Korhonen [26] reported that newly diagnosed pre-diabetes or diabetes per se is not associated with PAD, whereas long-term diabetes remains a well-established risk factor for PAD. A prospective cohort study by Joosten et al. [27] also found that 5 years or more of diabetes mellitus, dyslipidemia or hypertension was significantly associated with incidence of PAD.

Significantly higher proportions of vascular co morbidities, such as CAD and CVD, among those with PAD have been reported in many studies [5, 20, 28]. Compared with their non-PAD counterparts, a significantly higher proportion of males with PAD were also found to have ED. Several previous studies also have reported similar findings [29, 30]. Thus, ED has consequently become a significant co morbidity and predictor of asymptomatic PAD among males.

Claudication symptoms were found among only one-third of those with PAD. Low prevalence of claudication symptoms has also been reported by many other studies [8, 31–33]. The usefulness of intermittent claudication in identifying PAD is therefore limited.

Pedal pulses were categorized as being present, weak, or absent. Both pedal pulses were absent in almost two-thirds of PAD lower limbs. Thus, the usefulness of assessing pedal pulses is much greater than assessing claudication symptoms in the identification of PAD.

## Conclusions

This study revealed that the prevalence of PAD in Sri Lanka is lower than in Western countries. Significant associations of PAD were found with aging and other traditional cardiovascular risk factors. As all individuals found with PAD in this study were newly identified, PAD appears to be a hidden disease in the community and is an emerging public health concern in Sri Lanka, particularly with the aging population. There is an urgent need to take primary prevention measures to modify the risk factors of PAD. Incorporating public awareness activities about PAD, alongside health education and health promotion activities related to other non-communicable diseases, is one vital step towards addressing this need. Additional priorities beyond public awareness programs should include screening activities for early PAD identification, as well as the strengthening of healthcare facilities for managing PAD.

## Abbreviations

ABPI: Ankle Brachial Pressure Index
BMI: Body mass index
CAD: Coronary artery disease
CVD: Cerebrovascular disease
ED: Erectile dysfunction
PAD: Peripheral arterial disease
